# The effect of the mGlu8 receptor agonist, (S)-3,4-DCPG on acquisition and expression of morphine-induced conditioned place preference in male rats

**DOI:** 10.1186/s12993-021-00174-0

**Published:** 2021-02-21

**Authors:** Nazanin Kahvandi, Zahra Ebrahimi, Seyed Asaad Karimi, Siamak Shahidi, Iraj Salehi, Marzieh Naderishahab, Abdolrahman Sarihi

**Affiliations:** 1 Neurophysiology Research Center, School of Medicine, Hamadan University of Medical Sciences, Shahid Fahmideh Street, Hamadan, Iran; 2 Department of Neuroscience, School of Sciences and Advanced Technology in Medicine, Hamadan University of Medical Sciences, Hamadan, Iran; 3 Department of Physiology, Faculty of Medicine, Hamadan University of Medical Sciences, Hamadan, Iran

**Keywords:** Metabotropic glutamate receptor type 8, Nucleus accumbens, Conditioned place preference, Morphine, Rat

## Abstract

**Background:**

The nucleus accumbens (NAc) plays a principal role in drug reward. It has been reported that metabotropic glutamate receptors (mGlu receptors) play a key role in the rewarding pathway(s). Previous studies have shown the vast allocation of the different types of mGlu receptors, including mGlu8 receptors, in regions that are associated with opioid rewards, such as the NAc. The aim of the present study was to evaluate the role of mGlu8 receptors within the NAc in the acquisition and expression phases of morphine induced conditioned place preference (CPP). Adult male Wistar rats were bilaterally implanted by two cannulas' in the NAc and were evaluated in a CPP paradigm. Selective mGlu8 receptor allosteric agonist (S-3,4-DCPG) was administered at doses of 0.03, 0.3, and 3 μg/0.5 μL saline per side into the NAc on both sides during the 3 days of morphine (5 mg/kg) conditioning (acquisition) phase, or before place preference test, or post-conditioning (expression) phase of morphine-induced CPP.

**Results:**

The results revealed that intra-accumbal administration of S-3,4-DCPG (0.3 and 3 μg) markedly decreased the acquisition in a dose-dependent manner but had no effect on expression of morphine-induced CPP.

**Conclusions:**

The findings suggest that activation of mGlu8 receptors in the NAc dose-dependently blocks the establishment of morphine-induced CPP and reduces the rewarding properties of morphine which may be related to the glutamate activity into the NAc and in reward pathway(s). These data suggest that mGlu8 receptor may be involved in conditioned morphine reward.

## Background

Drug addiction is a complex neuro-behavioral disorder. The rewarding effects of drugs play a vital role in the acquisition and expression of substance abuse [1]. Dopaminergic [2] and opioidergic [3] mechanisms have been considered as the basic mechanisms of drug addiction for many years. Recently, it has become progressively clear that glutamate is involved in addiction and that glutamatergic neurotransmission may be responsible for brain plastic changes that lead to addictive behavior and relapse [4]. It is well established that glutamatergic neurotransmission in the mesocorticolimbic pathway is involved in different mechanisms of morphine dependence [5–7]. Glutamate is the most abundant excitatory neurotransmitter in the brain and glutamatergic transmission accounts for up to 70% of synaptic transmission in the central nervous system (CNS) [8]. Thus, there are glutamatergic projections and/or neurons expressing glutamate receptors in reward circuitry including ventral tegmental area (VTA), nucleus accumbens (NAc), amygdaloid complex and frontal cortex (FC) [9, 10].

The glutamate effects are mediated by ionotropic (iGlu) and metabotropic (mGlu). mGlu receptors are G-protein coupled receptors which have an important role in mediating glutamate neurotransmission in the CNS [11–13]. mGlu receptors are classified into three groups: Group I (mGlu1 and 5), Group II (mGlu2 and 3) and Group III (mGlu4, 6, 7, and 8) [14]. The Group III family normally inhibit glutamatergic neurotransmission and has been less studied due to the lack of appropriate selective drugs. However, this group of receptors are emerging as important contributors to stress-related disorders such as depression, anxiety, addiction, and schizophrenia [15, 16].

Recent studies have identified mGlu receptors as potential targets for the treatment of drug addiction. For instance it has been suggested that both subtypes of the group I mGlu receptors (mGlu1 and mGlu5 receptors) take part in the expression of morphine sensitization processes but mGlu1 is not involved in the expression of morphine withdrawal jumps in mice [17]. In their study, pretreatment with the mGlu1 receptor antagonist EMQMCM [3-ethyl-2-methyl-quinolin-6-yl-(4-methoxy-cyclohexyl)-methanone methanesulfonate] and the mGlu5 receptor antagonist MTEP ([(2-methyl-1,3-thiazol-4-yl) ethynyl] pyridine) was done. The mGlu5 receptor antagonist has been shown to block the development of cocaine- and morphine conditioned place preference (CPP) [18–20]. In Popik and Wro´bel work, during conditionings, mice were pretreated with placebo or MPEP (to investigate its effect on acquisition of morphine-induced CPP), 20 min before morphine or placebo injection. MPEP at 30, but not 10 mg/kg significantly inhibited the acquisition as well as expression of morphine induced CPP, but it neither produced place preference or aversion, nor affected locomotor activity of mice [18]. However, some studies have shown contradictory results regarding the role of mGlu5 receptor in morphine CPP. Others have even observed a potentiation in morphine CPP with MPEP as a mGlu5 receptor antagonists (timing of MPEP administration was during conditioning) [21–23]. Similarly, Group II mGlu receptors can regulate both reward processing and drug seeking [24] and in our previous work we showed that activation of mGlu2/3 receptors (during acquisition and expression) in the NAc dose-dependently blocked both the establishment and the maintenance of morphine-induced CPP [7].

mGlu group III has been suggested as a new target to treat substance use disorder [25]. However, the role of mGlu8 receptor in drug dependence is still not well investigated and very few studies have pointed out the role of mGlu8 receptor in the effects of drugs of abuse. Regarding, group III mGlu receptors, pretreatment microinjection of L-2-amino-4-phosphonobutyric acid (L-AP4), a non-selective agonist of group III mGlu receptors into the dorsal striatum reduced amphetamine or cocaine-induced hyperlocomotion in rats [26]. Backstrom and Hyytia showed that administration of mGlu8 receptor agonist S-(3,4)-DCPG during conditioning and extinction procedure reduced ethanol self-administration and cue-induced reinstatement of ethanol-seeking [27]. Also It is indicated that activation of mGlu4 receptor has an important effect on the rewarding properties of alcohol [28] and recently Zaniewska et al. showed that mGlu4 receptor activation reduces cocaine-, but not nicotine-induced locomotor sensitization [29]. Also, it has been shown that mGlu7 receptor orthosteric agonist, LSP2-9166 blocked morphine CPP expression and reinstatement after extinction [30].

Despite the above-mentioned results, further investigation is required to fully understand the role of group III mGlu receptors in the pathological process of drug addiction. There is also evidence that glutamate transmission in the NAc is involved in CPP induced by morphine, cocaine, or amphetamine [31]. A number of alterations in glutamatergic transmission occur within the NAc after withdrawal from chronic drug exposure [31]. NAc receives glutamatergic projections from the prefrontal cortex, thalamus, basolateral amygdala (BLA), and hippocampus [10, 32, 33]. The NAc is a major input structure of the basal ganglia and integrates information from cortical and limbic structures to mediate goal-directed behaviors [31]. Chronic exposure to several classes of drugs of abuse disrupts plasticity in NAc, allowing drug-associated cues to engender a pathologic motivation for drug seeking [31]. The NAc exhibits high expression of mGlu receptors, especially mGlu4, 5 and 8 receptors. The mGlu8 receptor mRNA expression is high in the NAc compared to other areas of the brain [34]. Previous evidences has shown that glutamate in the NAc plays an important role in opioid rewards, including its expression [7], extinction [35, 36], and reinstatement [35–37] of morphine-induced CPP. However, the precise role of mGlu8 receptor in morphine-induced CPP is unclear. Taken together, it seems that there is a type specificity in the role of mGlu receptors in different steps of drug abuse on the other hand the precise role of mGlu8 receptor in morphine-induced CPP is unclear. Therefore, the goal of the current study was to assess the involvement of intra-accumbal mGlu8 receptor in the acquisition and expression of morphine-induced CPP in male rats.

## Results

The dose–response for morphine on conditioned place in the CPP paradigm was examined and as previous studies, the minimum effective dose of morphine was 5 mg/Kg [5, 6].

### The effect of Intra-accumbal S-3,4-DCPG administration on the acquisition of morphine-induced CPP

**Fig. 1:**
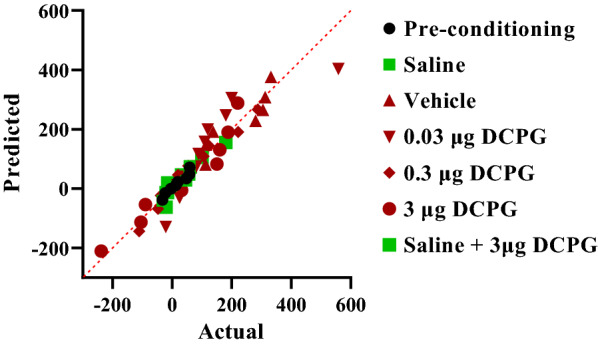
Q-Q plot for distribution of conditioning score data in the conditioning days. These data did not pass normality test. The data normality test was performed using Shapiro–Wilk test

**Fig. 2:**
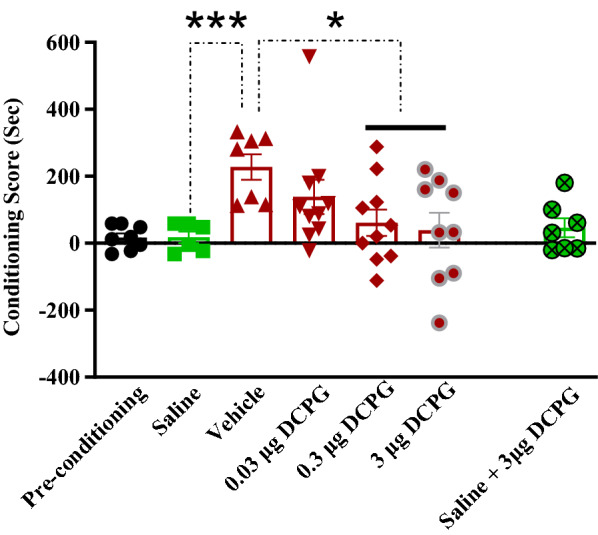
The effects of the administration of saline, as a vehicle, and different doses of S-3,4-DCPG, as a potent and selective mGlu8 agonist, (0.03, 0.3 and 3 μg/0.5 μL) into the nucleus accumbens (NAc), 5 min before the injection of morphine (5 mg/kg, SC) and administration of maximum dose of S-3,4-DCPG into the NAc region alone, in the conditioning days. Saline group received SC injection of saline instead of morphine during the acquisition phase. Bars represent mean ± S.E.M (Pre-conditioning; n = 8, Saline; n = 6, Vehicle; n = 7, different doses of S-3,4-DCPG (0.03 μg/0.5 μL; n = 10, 0.3 μg/0.5 μL; n = 10 and 3 μg/0.5 μL; n = 9 μg/0.5 μL). *P < 0.05, ***P < 0.001, Kruskal Wallis test followed by Dunn's multiple comparisons test

The data normality test was performed using Shapiro–Wilk test. Q-Q plot for distribution of CS data is shown in Fig. 1. These data did not pass normality test, so the Kruskal Wallis test was used. The analysis revealed that there was a significant difference between the saline SC injected (Saline, n = 6) and morphine SC injected + vehicle microinjection into the NAc control group (Vehicle, n = 7) (P = 0.0006, Fig. 2). The concurrent administration of intra-accumbal S-3,4-DCPG and systemic morphine during the acquisition period attenuated the rewarding attributes of morphine in the CPP paradigm in a dose dependent manner (P < 0.05, Fig. 2). In addition, administration of the highest dose of S-3,4-DCPG (3 μg/0.5 μL) alone did not affect the CS in saline-treated rats (Fig. 2).

### Effect of intra-accumbal S-3,4-DCPG administration on the expression of morphine-induced CPP

**Fig. 3:**
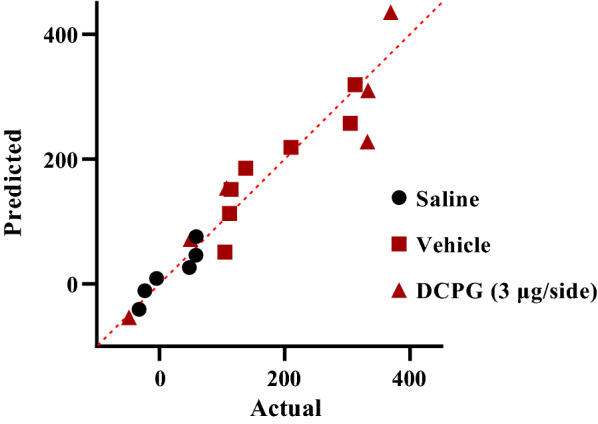
Q–Q plot for distribution of conditioning score data during post-conditioning (expression) phase. These data did not pass normality test. The data normality test was performed using Shapiro–Wilk test

**Fig. 4:**
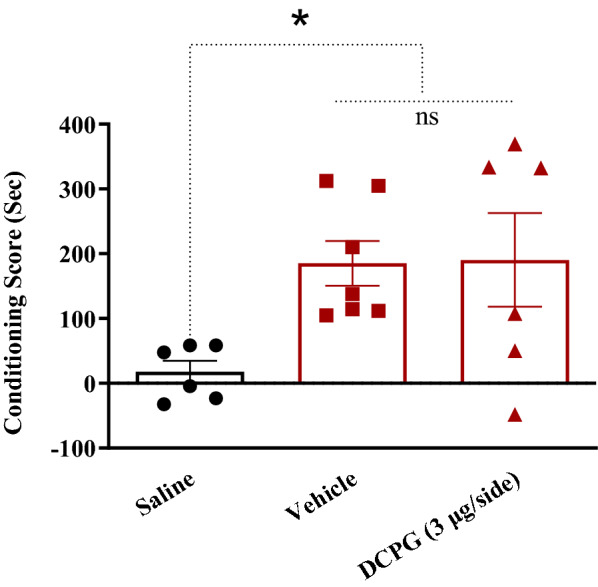
The effects of the administration of highest doses of S-3,4-DCPG, as a potent and selective mGlu8 agonist (3 μg/0.5 μL) into the nucleus accumbens (NAc) 5 min before the test on the post-conditioning day. Bars represent mean ± S.E.M (Saline; n = 6, Vehicle; n = 7, DCPG (3 μg/side); n = 6). ns: not significant, *p < 0.05. Kruskal Wallis test followed by Dunn's multiple comparisons test

The data normality test was performed using Shapiro–Wilk test. Q–Q plot for distribution of CS data during post-conditioning (expression) phase is shown in Fig. 3. These data did not pass normality test, so the Kruskal Wallis test was used. The analysis indicated that intra-accumbal administration of S-3,4-DCPG (3 μg/0.5 μL) had no effect on the expression of morphine-induced CPP in morphine treated animals (P > 0.05, Fig. 4). compared with the vehicle-control group. It means that S-3,4-DCPG (3 μg/0.5 μL) could not reverse or attenuate the morphine place preference.

### The effect of intra-accumbal S-3,4-DCPG administration during morphine-induced CPP on locomotor activity

**Fig. 5:**
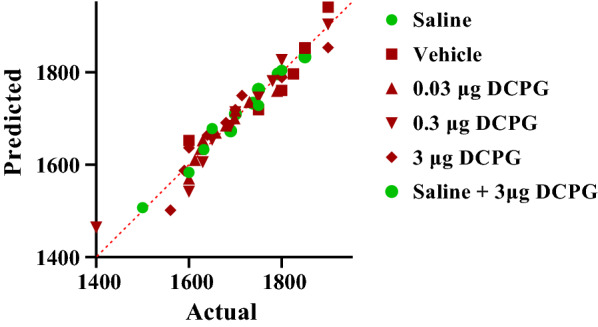
Q–Q plot for distribution of traveled distance data during post-conditioning or test day. These data passed normality test. The data normality test was performed using Shapiro–Wilk test

While our tracking system recorded the locomotion any time during the protocol (pre-conditioning, conditioning and post-conditioning phases of the CPP paradigm), first we checked locomotion of the rats in open field test 5 min after injection to 30 min later and find no significant differences between experimental groups. For summarizing the results, we only reported the locomotion of the experimental groups during the post-conditioning or test day. The data normality test was performed using Shapiro–Wilk test. Q-Q plot for distribution of traveled distance data during post-conditioning or test day is shown in Fig. 5. These data passed normality test, so the One-way ANOVA was used.

**Fig. 6:**
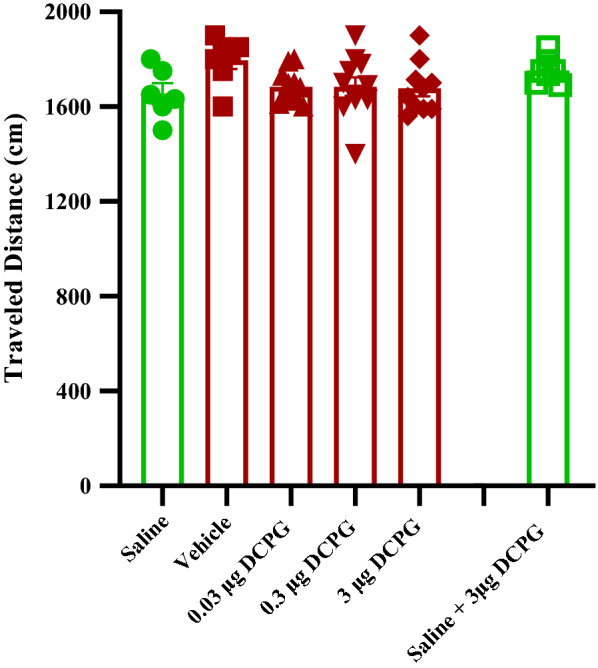
The effect of S-3,4-DCPG injection into the nucleus accumbens during morphine-induced conditioned place preference on locomotor activity. Bars represent mean ± S.E.M (Saline; n = 6, Vehicle; n = 7, different doses of S-3,4-DCPG (0.03 μg/0.5 μL; n = 11, 0.3 μg/0.5 μL; n = 11 and 3 μg/0.5 μL; n = 10 μg/0.5 μL)

One-way ANOVA followed by Newman-Keuls multiple comparison test [F (5, 45) = 0.1704, P = 0.9722; Fig. 6] indicated that S-3,4-DCPG did not change the traveled distance during the 10 min test period (on the post-conditioning or test day) in comparison with that of the vehicle control groups and saline SC administered group.

## Discussion

In the present study, the effect of S-3,4-DCPG as a selective mGlu8 receptor allosteric agonist within the NAc on development of morphine-induced CPP was investigated in rats. To our best knowledge, this is the first study which has examined the role of mGlu8 receptor within the NAc on morphine-induced CPP. Main findings of the present study can be expressed as: (a) bilateral intra-accumbal microinjection of S-3,4-DCPG dose-dependently reduced the acquisition of morphine-induced CPP, (b) after conditioning, intra-accumbal activation of mGlu8 receptor by S-3,4-DCPG at highest dose of 3 μg / 0.5 μL, it did not affect the expression of morphine-induced CPP in the rats, (c) administering the highest dose of S-3,4-DCPG into the NAc alone could not induce CPP, and (d) this drug did not affect locomotor activity. Brain regions that control locomotion, such as striatum, are enriched in group III mGlu receptors [38], indicating the possible role for these receptors in locomotor activity. Our data showed that S-3,4-DCPG did not affect locomotor activity. This indicates that mGlu8 receptors does not play a chief role in locomotor activity. On the contrary, it has been shown that other group III mGlu receptors (such as mGlu4 and mGlu7 receptors) are involved in the locomotion [28, 39, 40], that could be explained receptors type specificity in the role of mGlu receptors on locomotor activity, site specificity and by the animal species used and/or by the dose of agonists.

Since two decades, glutamatergic system has been involved in drug addiction. Among the components of the glutamatergic system, the presynaptic mGlu receptors have recently received much attention because of their role in glutamate release and regulation of glutamatergic responses. Up to present day only a few studies on the role of mGlu8 receptor in morphine dependence have been reported. Numerous findings confirm that the Group III mGlu receptors family plays an important role in drug addiction, regulating transmitter release and behavioral plasticity in the limbic system [41, 42].

Previous studies have shown that mGlu receptors are involved in the acquisition and expression of morphine-induced CPP [5, 6]. The NAc plays a crucial role in developing physical dependence on morphine [43]. Morphine eliminates the inhibitory effects of dopamine on glutamatergic inputs to the NAc neurons and enhances glutamatergic transmission to the NAc neurons, especially from the BLA to the NAc [44]. Other observations have reported that repeated exposure to opioids enhances the function of mGlu receptors and presynaptic stimulation of these receptors results in reduced glutamate release [45, 46]. It has also been shown that attenuation of glutamatergic neurotransmission through presynaptic mGlu receptor agonists is effective in suppressing drug craving and substance use [27].

The NAc can be subdivided into core and shell subregions [47] and a number of studies suggest that these subregions may be differentially involved in reward-related learning and locomotion [48–50]. Our data did not permit a direct investigation of the relative contribution of the two subregions. There is some evidences indicating the role of the NAc shell in the acquisition of CPP [31, 51]. However, there are also studies showing the involvement of the NAc Core too [31, 52–54]. In addition, some studies have shown the role of whole NAc in this issue including our previous studies [5, 55, 56]. On the other hand, there is no difference in case of mGlu8 receptor density between NAc shell and core [34]. Finally, based on the stereotaxic coordination, sizes of the NAc core and shell, the volume of injected drug and the time after injection to the end of the CPP session the diameter of spatio-temporal diffusion may include both parts of the NAc [57]. Based on the rat stereotaxic coordination the maximum length of the NAc (core and shell) ranged from 16 to 22 mm with a mean value (± SD) of 19.4 ± 1.5 mm and no asymmetry between right and left, it means a sphere with 0.8 to 1.1 mm radius. Even with a conservative estimation we can say that the spatio-temporal diffusion of our injected volume should include both part of the NAc. Therefore, we can’t consider one part effective and completely exclude the effects of drug on another part.

Findings from other studies confirm the findings of the current study regarding the inhibitory role of mGlu receptor activation in the NAc on drug induced CPP acquisition. Bahi showed that systemic injection of mGlu8 receptor agonist decreases voluntary ethanol intake and ethanol-induced CPP in C57BL/6J mice [58]. Several studies have suggested that mGlu8 receptors are involved in associative learning [59, 60]. Associative learning and synaptic long-term potentiation (LTP) depend on the same cellular mechanisms [61], which is consistent with our results regarding the inhibitory role of mGlu8 receptors in morphine induced CPP. It is possible that DCPG impair the formation of an association between the environmental cues in the morphine-paired compartment and the rewarding properties of the drug [62] without influencing the rewarding effects of morphine.

On the other hand, we found that mGlu8 receptor activation in the NAc has no effect on morphine reward after acquisition. Consistent with the results of the present study, findings from other studies showed that after conditioning, mGlu8 receptor activation had no effect on the expression of spatial conditioning relative to ethanol [58].

The systemic injection of cocaine decreases mGlu8 receptor protein levels in rat striatum. The decrease in mGlu8 receptor protein expression in the striatum may indicate a decrease in mGlu8 autoreceptors in corticostriatal terminals on the other hand as we know, presynaptic mGlu8 receptor inhibits glutamate release from corticostriatal terminals [63]. This transient deletion of inhibitory tone by mGlu8 receptor may be necessary to stimulate increased local release of glutamate and stimulate locomotor activity. Lack of presynaptic mGlu8 receptor can disrupt glutamatergic translocation in corticostriatal synapses, that is consistent with other mechanisms involved in behavioral responses to acute stimulation by cocaine [63].

In conclusion, the results of the current study revealed that intra-accumbal injection of mGlu8 receptor agonist (S-3,4-DCPG) in a dose-dependent manner reduced the acquisition while it had no effect on the expression of morphine-induced CPP. Taken together, the available evidences indicate an important modulatory rather than necessary role for mGluR8 in NAc based morphine reward. It can be proposed that the plasticity related to mGlu8 receptor downstream proteins during CPP of morphine could account for the behavioral response found by S-3,4-DCPG. Future studies are needed to characterize the specific mechanisms of action of mGlu8 receptor in acquisition and expression of morphine-induced place preference in rats.

## Methods

### Animal

10-week male Wistar rats weighing between 200 and 250 g were obtained from animal breeding colony of Hamadan University of Medical Sciences (Hamadan, Iran). They were maintained on 12/12 h light/dark cycle (light on at 7 AM) and had access to freely available food and water in their home cages (temperature 22 °C ± 2 °C). Wood shavings were used for the rats' bedding, which was changed daily. Lighting within cages during day hours should be held at lux ranges below thresholds of aversion for rats. We used LED lights (25 lx). In order to conduct behavioral tests, it is necessary to increase the level of light (approximately 210 lx). All experiments were performed in accordance with the guide for the Care and Use of Laboratory Animals (National Institutes of Health Publication, No. 80–23, revised 1996) and were approved by the institutional ethics committee at Hamadan University of Medical Sciences.

### Drugs

In the current study in order to conduct the experiments the following drugs were used as following: Morphine sulfate (Temad, Iran) was dissolved in normal saline (0.9% NaCl) (*S*)-3,4-Dicarboxyphenylglycine (S-3,4-DCPG) (Tocris, UK), a selective mGluR8 allosteric agonist, was also dissolved in normal saline (0.9% NaCl). It is worth mentioning that control and vehicle groups received saline.

### Stereotaxic surgery and drug administration

Subjects were anesthetized by Xylazine (10 mg/kg) and Ketamine (100 mg/kg) and placed in the stereotaxic apparatus (Stoelting, USA) with the incisor bar set at approximately 3.3 mm below horizontal zero in order to achieve a flat skull position. After an incision was made to expose the rat's skull, two points were determined and holed into the skull at stereotaxic coordinates of 1.4 ± 0.4 mm anterior to bregma, ± 1.5 mm lateral to the sagital suture, and 6.5 mm ventral from top of the skull according to the atlas of rat brain (Paxinos and Watson, 2007). Two guide cannulae (23-Gauge) with 12 mm length were inserted into the holes aiming at the NAc. The guiding cannulae were anchored with a jeweler's screw and the incision was closed with dental cement. After surgery, dummy inner cannulae that extended 0.5 mm beyond the guiding cannulae were inserted into the guiding cannulae and left in place until injections were made. All rats were allowed to recover for 1 week before starting the behavioral testing.

### Intra-accumbal injection

The rats were gently restrained by hand and the dummy cannulae were removed from the guiding cannulae. Drugs were directly injected into the NAc through the guiding cannulae using injector cannulae (30-gauge, 1 mm below the tip of the guiding cannula). Polyethylene tubing (PE-20) was used for attaching the injector cannula to the 1-μl Hamilton syringe. Doses of selective mGluR8 allosteric agonist, S-3,4-DCPG, (0.03, 0.3, and 3 μg/0.5 μL saline per side) were administered into the NAc. The injection volume into the NAc was 0.5 μL/side for all groups. Injections were made bilaterally over a 50 s period and the injection cannulae were left in the guiding cannulae for an additional 60 s in order to facilitate the diffusion of the drugs.

### Place conditioning apparatus and protocol

**Fig. 7:**
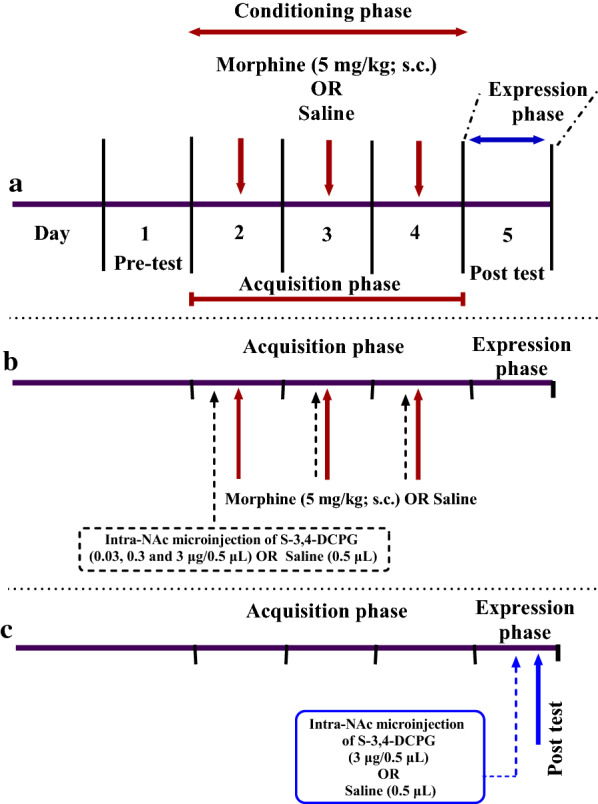
Graphical scheme to show behavioral protocol. **a** Acquisition and expression phases of Morphine-induced conditioned place preference (CPP) by injection of morphine (5 mg/kg; sc) during 3 days. **b** To investigate the role of mGlu8 in the nucleus accumbens (NAc) in the acquisition of morphine-induced CPP, the animals in the separate groups received S-3,4-DCPG, (0.03, 0.3, and 3 μg/0.5 μL saline) or saline into the NAc 5 min before the rats received morphine during acquisition phase. **c** To find out the role of mGlu8 in the expression of morphine -induced CPP, the animals received highest dose of S-3,4-DCPG (3 μg/0.5 μL saline) or Saline 5 min prior to CPP test

A three-compartment CPP apparatus was used in the experiments. The apparatus was comprised of three plexiglass compartments. The apparatus was divided into two equal-sized compartments (30 cm × 30 cm × 40 cm) with the third Sect. (30 cm × 15 cm × 40 cm) being the null section which connected the two equal-sized sections. Both compartments had white backgrounds with black stripes in different orientations (vertical vs. horizontal). To provide a tactile difference between the compartments, one of them had a smooth plastic floor, while the other compartment had a net-like floor (metal grid). In this apparatus, rats showed no consistent preference for either compartment. The CPP protocol has been previously described [5]. An unbiased allocation was used. Rats with a neutral preference (45–55% for either side) were randomly allocated their drug-paired side (unbiased allocation). In the CPP paradigm, the conditioning score (CS) and distance traveled were calculated based on a video recorded by a CCD camera with 30 frames per second (30 fps) resolution. The camera was placed 2 m above the CPP boxes and the locomotion tracking was measured by Maze Router homemade software, a video tracking system for automation of behavioral experiments. CPP paradigm took place for 5 continuous days, which consisted of three distinct phases: pre-conditioning, conditioning and post-conditioning [5, 6]. The schedule of the CPP paradigm is shown in Fig. 7a.

### Pre-conditioning phase

On day 1, each rat was separately placed in the apparatus for 10 min, with free access to all compartments. Animal movements were recorded by Maze Router tracking software and analyzed on the same day. Rats with any compartment preference were omitted from the experiment. 3 rats were excluded from this study due to compartment preference. Then rats were randomly assigned to one of the two groups (odd and even) for place conditioning [5].

### Conditioning phase

The morphine conditioning phase, also known as the acquisition phase, were conducted on days 2, 3 and 4. Each group of animals was randomly divided into even or odd. Odd animals received subcutaneous (SC) injection of saline and morphine (5 mg/kg) pairing in alternative morning and afternoon design with an interval of 6 h. The vice versa program for even animals was done. This phase consisted of a 3-day schedule of conditioning sessions. A total of six sessions (30 min each) was carried out. During these 3 conditioning days, in 3 sessions, animals were confined to one compartment, under the drug influence. During other three sessions, they were injected with saline while confined to the other compartment. Access to the other compartments was blocked on these days. Place preference was calculated as a preference score (time spent in drug paired zone-time spent in the saline paired zone) [5, 6]. During this phase, saline group animals received saline in both compartments during alternative morning and afternoon design with an interval of 6 h. Locomotor data were also collected throughout CPP testing in order to assess the development of behavioral sensitization.

### Post-conditioning phase

On the 5th day, the partition was removed and the rats could access the entire apparatus. The mean time spent for each rat in both compartments during a 10-min period was recorded. In order to calculate the conditioning score, the difference in the time spent for the drug- and saline-paired places was considered as the preference criteria. In the acquisition tests, no injection was given on the post-conditioning day.

## Experimental design

### The effect of intra-accumbal administration of mGluR8 allosteric agonist (S-3,4-DCPG) on the acquisition of morphine-induced CPP

To investigate the effects of mGlu8 agonist on the acquisition of morphine-induced CPP, bilaterally intra-accumbal injection of S-3,4-DCPG (0.03, 0.3, and 3 μg/0.5 μL) [64] was done 5 min prior to each morphine injection (5 mg/kg; SC) during the 3 days of conditioning phase (Fig. 7a, b). During this phase, a vehicle-control group received saline (0.5 μL) instead of S-3,4-DCPG into the NAc, prior to SC injection of morphine. Moreover, to rule out the possibility that S-3,4-DCPG administration alone had rewarding or aversive effects on the CPP, a separate group of rats received the highest doses (3 μg/0.5 μL) of S-3,4-DCPG prior to saline injection (1 mL/kg; SC) instead of morphine during the conditioning days. Saline group received saline SC injection instead of morphine during the conditioning phase.

### The effects of intra-accumbal S-3,4-DCPG injection on the expression of morphine-induced CPP

In order to examine the effects of the highest dose of S-3,4-DCPG (3 μg/ 0.5 μL) on the expression of morphine-induced CPP, the rats were bilaterally given S-3,4-DCPG or saline into the NAc 5 min prior to CPP test (Fig. 7c). In addition, a control vehicle group received saline (0.5 μL) through the NAc instead of S-3,4-DCPG before CPP test on post-conditioning phase. The saline group received saline instead of morphine during the conditioning phase.

### Locomotor activity measurement

The locomotor activity of each rat was recorded using the locomotion tracking apparatus by a video tracking system (Router maze software). In these experiments, the total distance traveled (in centimeters) by each rat was measured in pre- and post-tests for all groups.

### Histology

**Fig. 8:**
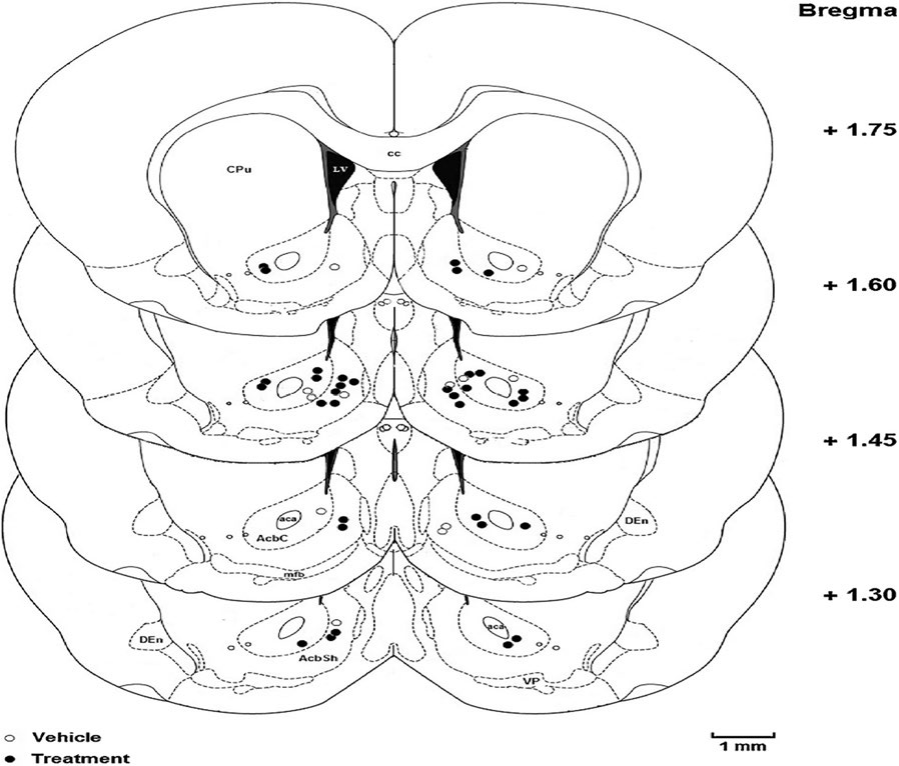
Histology. Schematic illustration of rat brain coronal sections showing the approximate location of the NAc injection sites. The numbers show the anterior–posterior coordinates relative to bregma. Atlas plates were adapted from Paxinos and Watson (Paxinos and Watson, 2007). Scale is 1 mm. It should be noted that some points are completely or partially overlapped (Vehicle; n = 31, Treatment; n = 44)

After behavioral testing, all the rats implanted with injection cannulae were deeply anesthetized with Ketamine and Xylazine. They were then transcardially perfused with 0.9% saline and then a 10% formalin solution. The brains were removed, blocked, and cut coronally (by using a vibratome) in 50 μm sections through to the cannulae. All the rats with cannula placement 1 mm distant from the intended injection site were removed from the data (Fig. 8). It should be noted that some points are completely or partially overlapped.

### Statistics

Data were processed by commercially available software GraphPad Prism® 8.0.2. The data normality test was performed using Shapiro–Wilk test. If the data passed normality test (Shapiro–Wilk test greater than 0.05), we used one-way analysis of variance (ANOVA) followed by post hoc analysis (Newman–Keuls multiple comparison test). But If the data did not pass normality test (Shapiro–Wilk test less than 0.05), Kruskal Wallis test was used followed by Dunn's multiple comparisons test. The Kruskal Wallis test is the non parametric alternative to the one-way ANOVA. Multiple student's t-test was used to compare pre-conditioning with saline or highest dose of S-3,4-DCPG (3 μg/0.5 μL). P-values less than 0.05 (P < 0.05) were considered to be statistically significant.

## Data Availability

The data are available for any scientific use with kind permission.
